# Prediction of all-cause in-hospital mortality after ICU admission and 1-year mortality after discharge of patients with acute kidney injury

**DOI:** 10.1080/0886022X.2025.2562445

**Published:** 2025-09-29

**Authors:** Lei Zhong, Li-ying Han, Yan-fang Wu, Hui-yan Huang, Zhuo-yan Wang

**Affiliations:** ^a^ Clinical laboratory, Tongxiang Hospital of Traditional Chinese Medicine, Jiaxing, Zhejiang, China; ^b^ Department of Hematology, The First People’s Hospital of Fuyang Hangzhou, Hangzhou, Zhejiang, China; ^c^ Health Management Center, Zhejiang Provincial People’s Hospital, Hangzhou, Zhejiang, China

**Keywords:** Prediction, AKI, mortality, XGBoost

## Abstract

Acute kidney injury (AKI) is a common comorbidity for in-hospital patients. This study aimed to develop dual prediction models for in-hospital and 1-year post-discharge mortality in patients with AKI, with a focus on identifying novel immunological risk factors. 7,937 patients (1,567 in-hospital deaths; 6,370 discharged) were selected from the Medical Information Mart for Intensive Care (MIMIC-IV) database based on serum creatinine and urine output criteria. Forty-two features were selected using least absolute shrinkage and selection operator (LASSO) regression and were used to construct prediction models. eXtreme Gradient Boosting (XGBoost) showed the best performance for in-hospital mortality prediction (training area under the curve (AUC) = 0.833; test AUC = 0.755). Kaplan-Meier curve and log-rank test revealed significant differences in 1-year mortality rates between patients without AKI and patients with AKI stage greater than 2 and those patients who did not recover from AKI at discharge showing a higher 1-year mortality risk (Hazard Ratio (HR) = 1.2, 95% Confidence Interval (CI) = 1.1–1.2). Among the five independent risk factors associated with 1-year mortality identified using univariate Cox regression, two were immune-related cells: neutrophils (HR = 1.01, 95% CI: 1.00–1.01), and neutrophil-to-lymphocyte ratio (HR = 1.01, 95% CI: 1.00–1.02). In conclusion, XGBoost demonstrated the highest predictive performance for in-hospital mortality, with simplified acute physiology score II emerging as the most significant variable.

## 1. Introduction

Acute kidney injury (AKI) is defined as the rapid loss of kidney function and is commonly diagnosed based on serum creatinine (SCr) and urine output (UO) measurements. According to the 2012 Kidney Disease Improving Global Outcomes (KDIGO) guidelines, AKI can be further subdivided into stages based on the severity of dysfunction [1]. AKI is a common comorbidity in hospitalized patients and is associated with poor prognosis and increased mortality compared to patients without AKI. Its global prevalence among hospitalized patients ranges from 10% to 36.6% [2,3]. In the intensive care unit (ICU), AKI contributes substantially to morbidity and mortality rates, and in severe cases, may necessitate renal replacement therapy or lead to cardiovascular complications, hospital readmission, or recurrent AKI episodes [4–7].

Growing interest have focused on early AKI detection and prevention through the machine learning-based electronic alert systems and the identification of reliable biomarkers [3,8]. However, these approaches face challenges including cost, limited interpretability for clinicians, and inconsistent predictive accuracy of biomarkers and models [9,10]. There is increasing recognition that AKI is a potential gateway to chronic kidney disease, demanding deeper mechanistic understanding and more effective therapeutic strategies [11,12]. Although the full pathogenesis of AKI remains unclear, it is well established that tubular epithelial cells participate in inflammatory cascades by producing pro-inflammatory cytokines and chemokines [13,14]. Immune cells play dual roles—macrophages, for example, can be pro-inflammatory during early injury and anti-inflammatory during repair due to their plasticity. Regulatory T cells (Tregs) within the kidney have also shown protective effects against renal injury [15–17]. The interplay between tubular cells, immune cells, and their mediators can create a malicious cycle, potential contributing to progressive renal dysfunction and poor clinical outcomes.

Machine learning (ML) has been increasingly adopted in medicine to assist clinicians, bolstered by large-scale public databases such as the Medical Information Mart for Intensive Care (MIMIC) and Surveillance, Epidemiology, and End Results (SEER) databases [18–21]. Moreover, studies have demonstrated the vast scope of the use of machine learning for AKI, including risk factor selection [22,23], early detection of AKI onset [24,25], prediction of mortality [26–28], and performance comparison of machine learning methods [29,30].

Despite progress, most existing machine learning models for AKI have focused on in-hospital mortality, with limited attention to long-term post-discharge outcomes. Additionally, only a few studies have incorporated immune cells as potential biomarkers of AKI progression [31–33]. To address these gaps, we utilized the MIMIC-IV database to develop the first integrated predictive framework targeting both in-hospital and 1-year post-discharge mortality in patients with AKI and systematically evaluated immune biomarkers alongside traditional predictors using Cox-LASSO regression. In this study, we aimed to build a machine learning-based model for predicting in-hospital mortality in patients with AKI using eight algorithms, selecting the best-performing model. As a secondary outcome, we analyzed 1-year post-discharge mortality using laboratory tests collected 12 h before discharge, rather than those from the first 24 h of ICU admission, to improve predictive accuracy.

## 2. Methods

### 2.1. Data source

Patient data were extracted from the MIMIC-IV 2.0 database, containing records of patients admitted to the critical care units of the Beth Israel Deaconess Medical Center in Boston. Our dataset included patients admitted between 2008 and 2019 [34,35]. Access to MIMIC-IV 2.0 was obtained by completing the HIPPA assessment *via* PhysioNet (ID: 55485857).

### 2.2. Data preprocessing

There were 180,733 unique patients in the MIMIC-IV database, and some had multiple admissions. We selected patients diagnosed with AKI based on two parameters SCr and UO. According to the Kidney Disease: Improving Global Outcomes (KDIGO) guidelines, the inclusion criteria for AKI were patients with SCr level increased by 0.3 mg/dL within 48 h, an increase in SCr over 1.5 times the baseline occurring within the prior 7 days, or UO less than 0.5 mL/kg/h for 6 h. We further categorized patients with AKI into three stages according to the following criterions: Stage 1: SCr was 1.5–1.9 times the baseline, SCr increased over 0.3 mg/dL, or the UO was less than 0.5 mL/kg/h for 6–12 h; Stage 2: SCr was 2.0–2.9 times the baseline or the UO was less than 0.5 mL/kg/h for over 12 h; And Stage 3: The SCr was 3 times the baseline, SCr increased over 4.0 mg/dL, or the UO was less than 0.5 mL/kg/h for over 24 h. In the present study, the baseline SCr level was defined as the initial SCr level measured within 24 h of ICU admission.

We extracted the first measurement of vital signs and laboratory tests for hospitalized patients who were admitted to the ICU within 24 h, and the same measurements for patients 12 h prior to discharge. The laboratory tests included hematocrit, hemoglobin, platelets, white blood cell (WBC), albumin, globulin, total protein, anion gap, bicarbonate, blood urea nitrogen, calcium, chloride, creatinine, glucose, sodium, potassium, basophils, eosinophils, lymphocytes, monocytes, neutrophils, atypical lymphocytes, bands, immature granulocytes, metamyelocytes, nucleated red blood cell (NCBC), d-dimer, fibrinogen, thrombin, international normalized ratio (INR), Prothrombin time (PT), partial thromboplastin time (PTT), alanine aminotransferase (ALT), alkaline phosphatase (ALP), aspartate transaminase (AST), amylase, bilirubin direct, bilirubin indirect, creatine kinase creatine phosphokinase (CK-CKP), creatine kinase isoenzymes-MB (CK-MB), γ-glutamyl transpeptadase (GTT), and lactate dehydrogenase (LDH). Furthermore, the Simplified Acute Physiology Score II (SAPS II), which reflects the severity of illness at admission, was included in the analysis. The SAPS II score comprises 17 variables, with each variable assigned a score between 0 and 26, resulting in a total score between 0 and 163. A higher SAPS II score indicated a higher risk of mortality. For patients with multiple admissions for AKI, only the first admission was included. Furthermore, we extracted most of the laboratory tests by applying the codes from the shared MIMIC Code Repository in GitHub from MIT, and the rest of the laboratory test values through customized PostgreSQL scripts.

Of the 180,733 patients admitted to the hospital, we screened 31,533 patients with AKI based on the criteria in the KDIGO guidelines. In addition to the AKI criteria, we excluded patients who were under 18 years old and over 90 years old; ICU length of stay shorter than 1-day; patients lacking data regarding neutrophil, monocyte, basophil, eosinophil, and platelet levels; and those with over 20% missing values on demographics, vital signs, and other laboratory tests. The mean value of a particular variable was imputed for the remaining missing values. The number of patients decreased to 24,675, and we extracted the first admission record to the ICU for each patient and filtered out laboratory tests within 24 h in the ICU. The final sample size for the prediction model was 7937. Among these, 1567 patients died during hospitalization and 6370 patients were successfully discharged. Patients discharged from hospital were included in the survival analysis.

### 2.3. Feature selection

The data were divided into training and test sets in a 1:1 ratio. Due to the nature of our unbalanced data, we applied random oversampling examples (ROSE) to the training set to generate a dataset with the same number of patients who died (*n* = 1984) or survived (*n* = 1984) during hospitalization. Using the adjusted balanced training set, we obtained 3968 patients and 45 corresponding features, including demographics, vital signs, various laboratory tests, and SAPS II scores. In addition, we explored the association between in-hospital mortality and several immune cell types, including basophil, eosinophils, lymphocytes, monocytes and neutrophils. LASSO regression was then applied for feature selection in the training set. The outcome variable was patient survival during hospitalization. The minimum lambda value that exhibited optimal performance was selected for the final LASSO regression to achieve better results. Variables with non-zero coefficients were selected for further model construction (*n* = 42).

### 2.4. Model construction

Eight machine learning algorithms were applied to the LASSO-selected features to predict in-hospital survival among patients with AKI. These included logistic regression, random forest, XGBoost, naïve Bayes, decision trees, bagging, support vector machine (SVM), and AdaBoost. Some methods enabled the visualization of variable importance in the form of trees and bar plots. With proper hyperparameter tuning and 5-fold cross validation, we obtained the optimized parameters from the training set and applied them to the test sets.

### 2.5. Survival analysis

For the 6370 patients with AKI who survived during hospitalization, we extracted their survival status after discharge and the date of death if the patient died from the follow-up information provided by the MIMIC-IV. The cutoff time point in our study was 365 days; therefore, we performed a 1-year survival analysis of patients who were successfully discharged from the hospital. Laboratory tests were performed 12 h prior to patient discharge (*n* = 5305). The patients were categorized into groups based on their AKI stage before discharge. We first compared the survival times of patients with different AKI stages at discharge using the Kaplan-Meier curve.

In addition, we performed Cox-LASSO regression to filter out features that were survival-related to discharged patients. First, we split the data into training and test sets of equal size. Moreover, we stratified the training set into five smaller sets while performing Cox-LASSO regression for cross-validation to achieve better performance. The best lambda value was selected and used to evaluate the models with the hyperparameters in the test set. Harrell’s C-index was used as an evaluation metric. The best lambda value was then selected from cross-validation and adapted to the training set. We then extracted variables with non-zero coefficients and ran a univariate Cox regression again to specify their impact on patient survival. Univariate Cox regression combined with multivariate Cox regression was used to select individual risk factors for 1-year survival.

### 2.6. Statistical analysis and software

Most data extraction and filtration processes were performed using PostgreSQL 15. All statistical analyses were performed using R Studio (version 4.3.1). The training and test sets were split in the ratio of 1:1 at a predetermined random seed, and the missing values in the training set were imputed using Multivariate Imputations by Chained Equations (MICE), implemented with ‘mice’ package, with the following parameter: *m* = 5, maxit = 30, meth = ‘pmm’. The R package ‘ROSE’ was utilized for the majority class under-sampling and minority class oversampling to solve the issue of unbalanced data in the training set. The hyperparameter tuning by random search and 5-fold cross validation were carried with the package ‘caret’. Furthermore, the evaluation metric for all the methods in this study was the area under the curve (AUC) for training and test sets. A receiver operating characteristic (ROC) curve was used to visualize model performance. ‘pROC’ was used in R to generate AUCs and plotted ROCs. The package ‘glmnet’ was employed for 5-fold cross validation while applying lasso regression for feature selection. The log-rank test was used to compare differences among the survival groups.

## 3. Results

### 3.1. Data statistics

**Figure 1.**
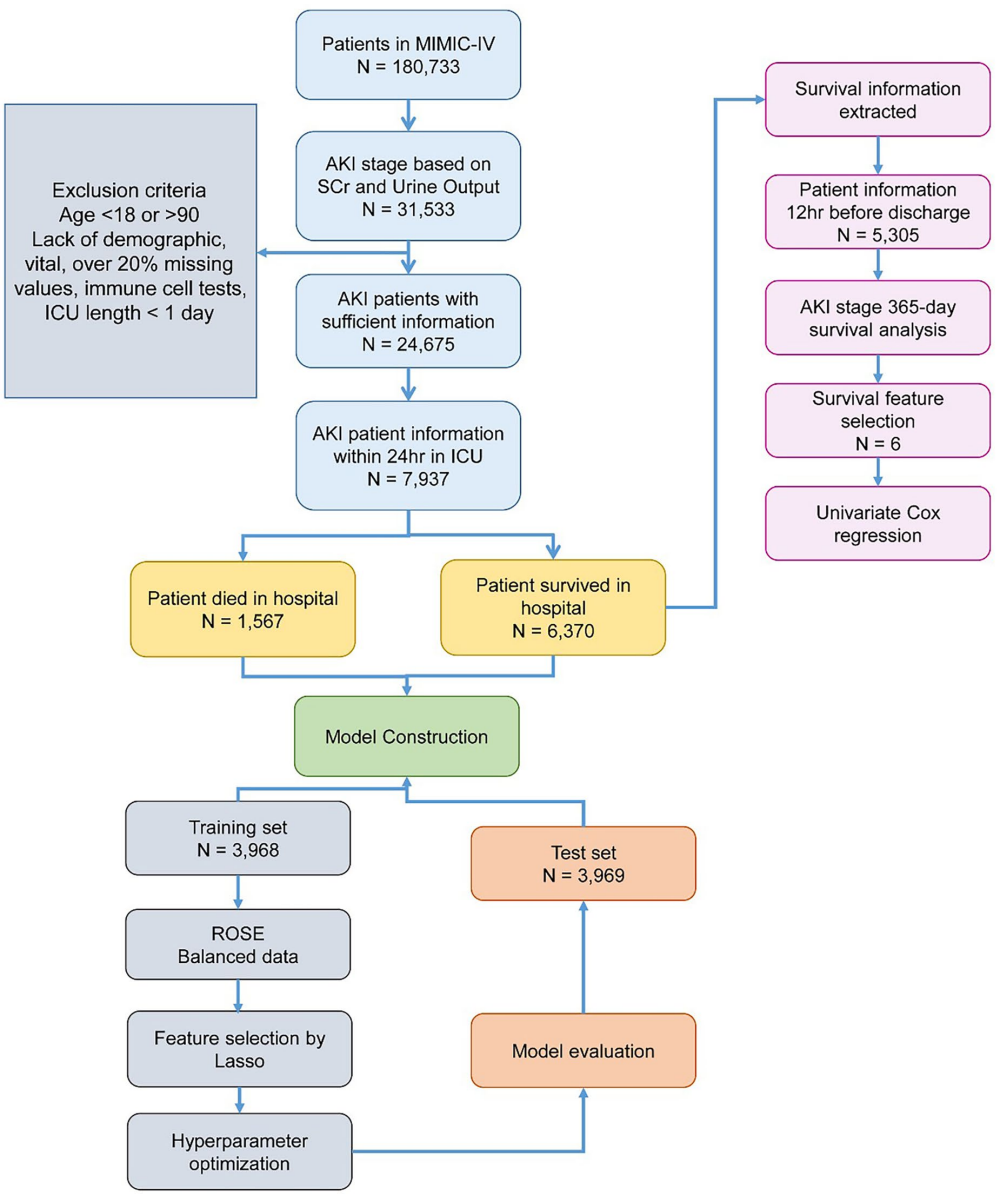
Study flowchart.

The workflow of this study is illustrated in Figure 1, and general patient characteristics are summarized in Table 1. Patients were categorized into two groups: those who survived and those who died during hospitalization. The mean age of survivors was 65.6 years old, which was 3.2 years younger than that of the patients who died during hospitalization. Most patients in both groups were diagnosed with Stage 2 AKI, while Stage 3 AKI was the least. As expected, patients who died had higher SAPS II scores than those who survived, according to the mean and median scores (Table 1). The ratios of immune cells to lymphocytes were calculated and the results showed that the ratios in patients who survived were lower than those in patients who did not survive.

**Table 1.** Summary statistics of in-hospital patients with acute kidney injury (AKI).

	0 (Survived)	1 (Death)	*p* Value
	(*N* = 6,370)	(*N* = 1,567)	
AKI_stage (%)			<0.001
1	2,207 (34.6)	346 (22.1)	
2	3,372 (52.9)	660 (42.1)	
3	791 (12.4)	561 (35.8)	
Sex = M (%)	3,881 (60.9)	913 (58.3)	0.057
Race (%)			<0.001
ASIAN	107 (1.7)	38 (2.4)	
BLACK	371 (5.8)	85 (5.4)	
HISPANIC	188 (3.0)	30 (1.9)	
OTHER	1,459 (22.9)	551 (35.2)	
WHITE	4,245 (66.6)	863 (55.1)	
Age (mean (*SD*))	65.55 (15.98)	68.78 (15.50)	<0.001
Heart rate, bpm (mean (*SD*))	113.46 (22.88)	130.49 (25.99)	<0.001
Systolic Blood Pressure (SBP), mmhg (mean (*SD*))	161.73 (26.12)	167.14 (32.87)	<0.001
Diastolic Blood Pressure (DBP), mmhg (mean (*SD*))	102.35 (25.65)	104.99 (28.76)	<0.001
Myelin basic protein (MBP), mmhg (mean (*SD*))	126.25 (42.95)	137.19 (52.37)	<0.001
SBP noninvasive, mmhg (mean (*SD*))	153.61 (25.51)	153.53 (28.95)	0.917
DBP noninvasive, mmhg (mean (*SD*))	98.26 (24.04)	98.49 (26.07)	0.742
MBP noninvasive, mmhg (mean (*SD*))	109.88 (23.89)	110.56 (26.15)	0.323
Respiratory rate, insp/min (mean (*SD*))	33.03 (8.45)	36.71 (8.61)	<0.001
Temperature, °C(mean (*SD*))	37.78 (0.80)	38.12 (1.10)	<0.001
Simplified Acute Physiology Score II (mean (*SD*))	39.06 (13.31)	54.81 (15.82)	<0.001
Hematocrit, % (mean (*SD*))	35.96 (6.18)	35.52 (7.13)	0.013
Hemoglobin, g/dL (mean (*SD*))	11.85 (2.13)	11.55 (2.36)	<0.001
Platelets, K/μL (mean (*SD*))	220.81 (107.22)	216.16 (121.65)	0.134
White blood cell, K/μL (mean (*SD*))	16.12 (9.91)	18.72 (19.77)	<0.001
Anion gap, mEq/L (mean (*SD*))	16.18 (4.88)	20.24 (6.83)	<0.001
Bicarbonate, mEq/L (mean (*SD*))	24.38 (3.99)	22.82 (5.09)	<0.001
Blood urea nitrogen, mg/dL (mean (*SD*))	27.31 (21.46)	42.63 (29.58)	<0.001
Calcium, mg/dL (mean (*SD*))	8.52 (0.79)	8.59 (1.12)	0.002
Chloride, mEq/L (mean (*SD*))	106.66 (5.99)	106.38 (7.76)	0.129
Creatinine, mg/dL (mean (*SD*))	1.64 (1.87)	2.29 (1.82)	<0.001
Glucose, mg/dL (mean (*SD*))	166.97 (104.37)	206.48 (117.88)	<0.001
Sodium, mEq/L (mean (*SD*))	139.84 (4.61)	140.84 (6.37)	<0.001
Potassium, mEq/L (mean (*SD*))	4.66 (0.81)	4.93 (1.00)	<0.001
Absolute Basophils, K/μL (mean (*SD*))	1.93 (5.92)	2.44 (8.48)	0.005
Absolute Eosinophils, K/μL (mean (*SD*))	5.86 (21.62)	5.85 (18.14)	0.976
Absolute Lymphocytes, K/μL (mean (*SD*))	81.30 (232.40)	125.36 (1,158.83)	0.005
Absolute Monocytes, K/μL (mean (*SD*))	29.63 (49.62)	41.70 (72.04)	<0.001
Absolute Neutrophils, K/μL (mean (*SD*))	561.88 (713.28)	737.87 (849.09)	<0.001
Basophils, % (mean (*SD*))	0.31 (0.30)	0.28 (0.41)	0.002
Eosinophils, % (mean (*SD*))	0.99 (1.33)	0.81 (1.58)	<0.001
Lymphocytes, % (mean (*SD*))	13.93 (9.44)	12.02 (12.93)	<0.001
Monocytes, % (mean (*SD*))	5.12 (3.48)	5.66 (5.10)	<0.001
Neutrophils, % (mean (*SD*))	79.61 (11.02)	79.78 (16.28)	0.618
Prothrombin time, sec (mean (*SD*))	16.96 (9.95)	22.50 (17.58)	<0.001
Partial thromboplastin time, sec (mean (*SD*))	42.87 (29.08)	56.07 (37.77)	<0.001
Neutrophil-to-lymphocyte ratio (mean (*SD*))	10.05 (11.20)	14.32 (15.90)	<0.001
Monocyte-to-lymphocyte ratio (mean (*SD*))	0.57 (0.65)	0.84 (1.02)	<0.001
Basophils-to-lymphocytes ratio (mean (*SD*))	0.03 (0.04)	0.03 (0.06)	<0.001
Eosinophils-to-lymphocytes ratio (mean (*SD*))	0.08 (0.14)	0.09 (0.18)	0.066
Systemic immune-inflammation (mean (*SD*))	2,374.75 (3,513.89)	3,155.87 (4,122.08)	<0.001

### 3.2. Feature selection

**Figure 2.**
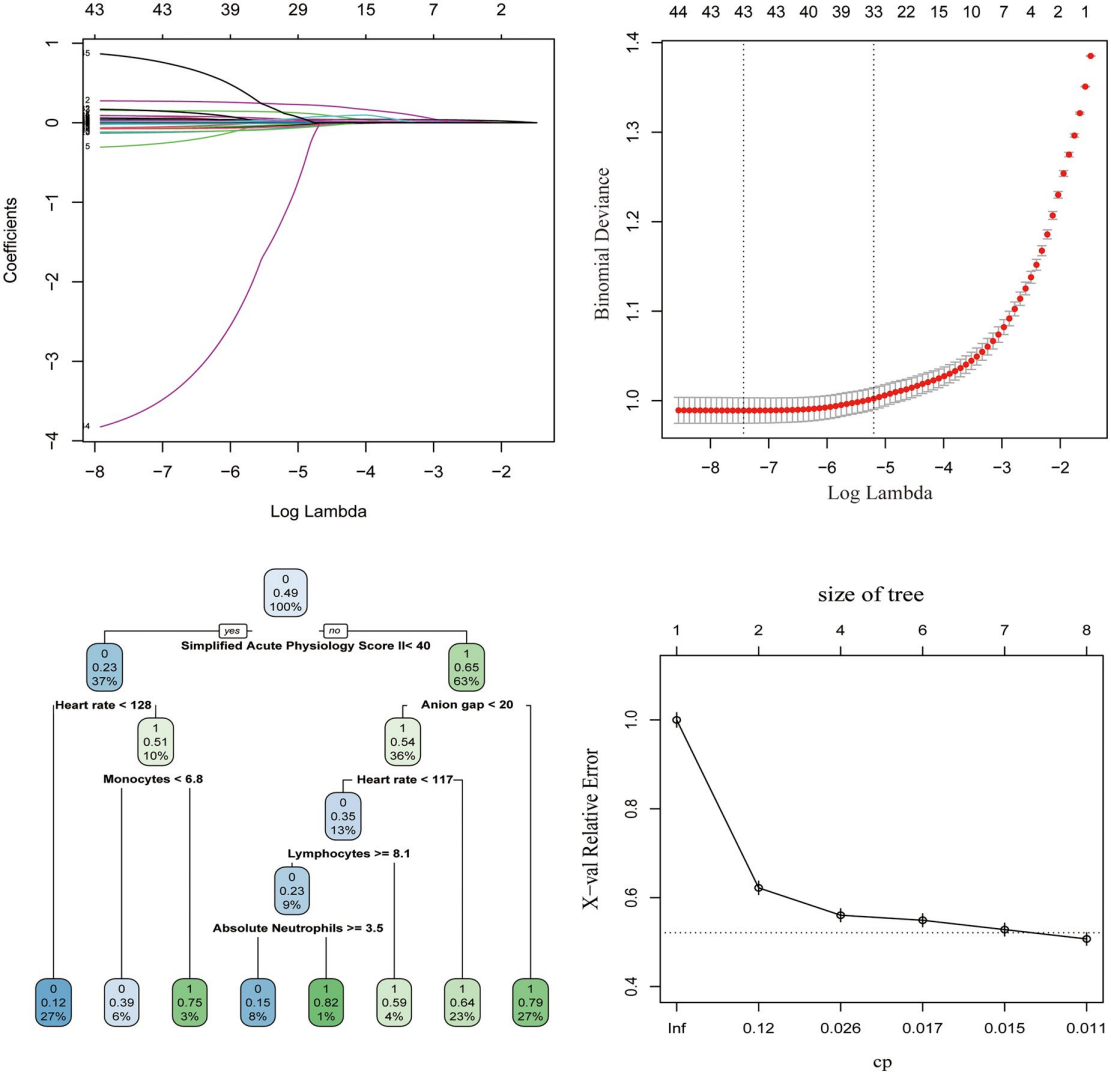
LASSO regression and model construction. (A) Plot displaying the shrinkage of all variables in LASSO regression with listed lambda values. (B) Plot indicating the minimum lambda values with corresponding number of features obtained using LASSO regression. (C) Random forest tree indicating the important variables. (D) Line graph indicating the error change according to the size of trees in random Forest.

The coefficients of the LASSO regression are shown in Figure 2A. The corresponding coefficients did not qualify for further analysis; therefore, we needed a threshold to determine the number of coefficients and the coefficients that were useful to retain. As shown in Figure 2B, the number of coefficients remained and the corresponding value of lambda, which is the dashed line on the left side, indicates that 42 features were selected. The lambda value was evaluated using 10-fold cross-validation with the training set.

### 3.3. Model evaluation

**Figure 3.**
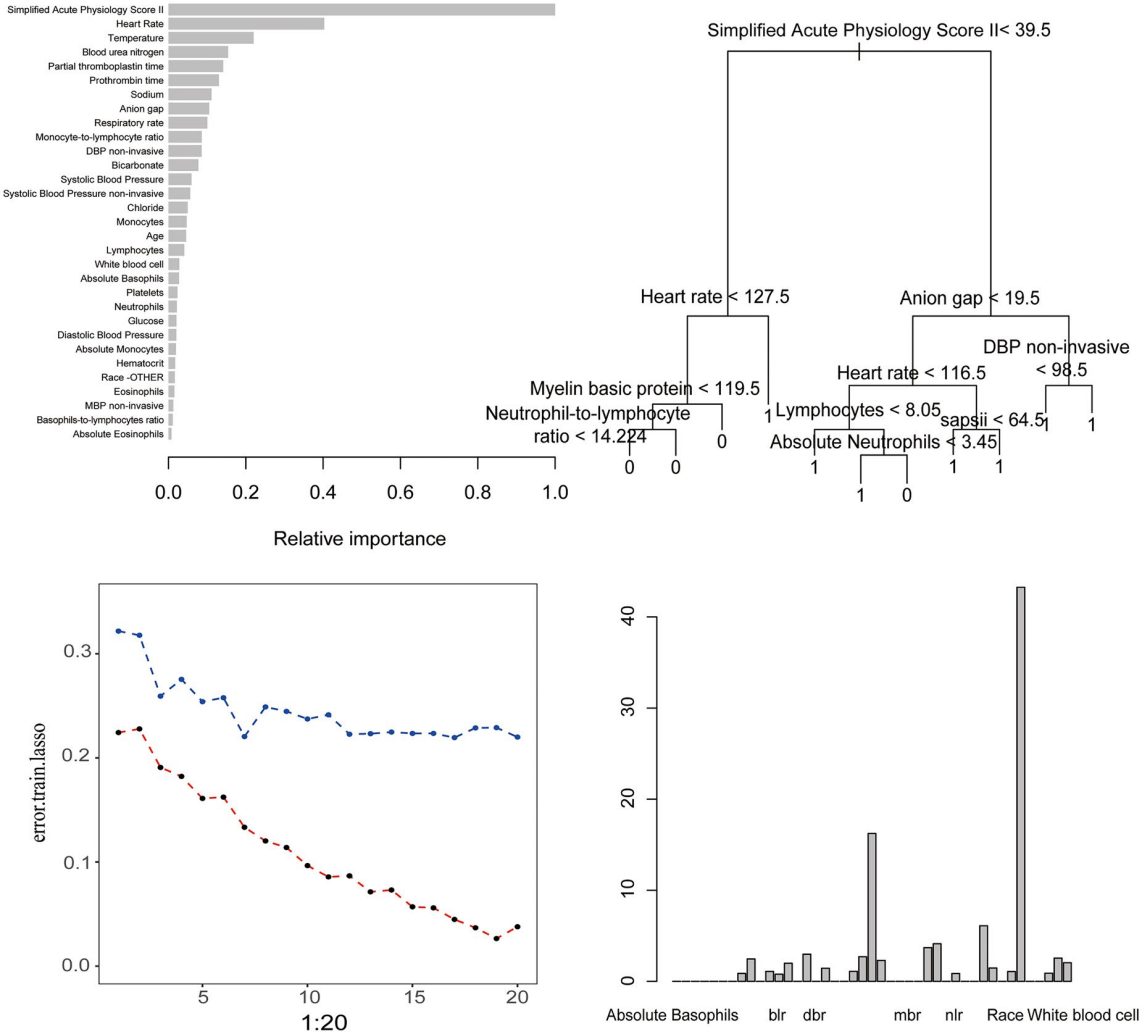
Feature importance analysis across predictive models. (A) Variable importance scores from the XGBoost model. (B) Decision tree structure showing node composition (SAPSII: Simplified acute Physiology score II). (C) AdaBoost error rates across sequential iterations. (D) Key predictors identified by AdaBoost: Basophils-to-lymphocytes ratio (BLR), diastolic blood pressure (DBP), myelin basic protein (MBP), and neutrophil-to-lymphocyte ratio (NLR).

A class-balanced training set was obtained using the ROSE algorithm. In this study, we used eight machine learning algorithms to predict the in-hospital mortality in patients with AKI. The first model we tested was a logistic regression model. We trained our coefficients on the training set and evaluated their performance on the test set. The second algorithm used was random forest, which provided variables that played significant roles in making decisions when building the model. The random forest model provides a decision tree that denoted each important feature that determined the likelihood of patient survival. For example, the SAPS II score was the most critical feature, which was the root node of the decision tree. The internal nodes were the rules for each branch, such as whether or not the heart rate exceeded 128 beats/min, and decisions were made based on the classification of each leaf node. Thus, the leaf nodes were the final decisions regarding whether the patient died or survived based on previous decisions. Patients with a SAPS II score less than 40 were more likely to survive, and vice versa (Figure 2C). Furthermore, we plotted the change in error as the size of the trees in each iteration changed in Figure 2D. In the context of pruning, the leftmost value for which the mean is below the horizontal line is often a favorable option. The next algorithm applied was XGBoost, and multiple combinations of hyperparameters were adjusted using the XGBoost algorithm to obtain a bar plot of the variable importance of the model (Figure 3A). The variable importance from XGBoost was consistent with that of the random forest, in which the SAPS II score played the most essential role in the dataset. Because the XGBoost model was constructed in a binary logistic regression form, the importance was calculated by the gain from splitting the trees, where the higher the gain score, the more important the contribution of the feature to the model.

The following models were constructed: naïve Bayes, decision tree, bagging, support vector machine (SVM), and AdaBoost. Figure 3B shows a tree from the decision tree model with a pattern similar to that of random forest tree. Again, the SAPS II score was the root node and therefore the most important feature. This was followed by two secondly important features: heart rate and anion gap. Subsequent features were predominantly immune-related indices, highlighting the significant role of immunological markers in the analysis. Unfortunately, the naïve Bayes, bagging, or SVM models were not visualized. For AdaBoost, we first set 20 different numbers of iterations for boosting and drew a line graph indicating the errors in the training and test sets to select the optimal number of iterations (Figure 3C). The line plot shows that the error in the training set decreased as the number of iterations increased, whereas the error in the test set reached its minimum at the 7^th^ iteration. We then evaluated the model at the 7^th^ iteration for both the training and test sets. A bar plot of variable importance is shown in Figure 3D, which represents the relative importance of each variable in the AdabBoost classification task. In AdaBoost, the variable importance considered the gain in the Gini index given by a variable in a tree and its weight. Similar to the rankings at other variable importance level, the most important variable in AdaBoost was the SAPS II score, followed by the prothrombin time and hematocrit.

**Figure 4.**
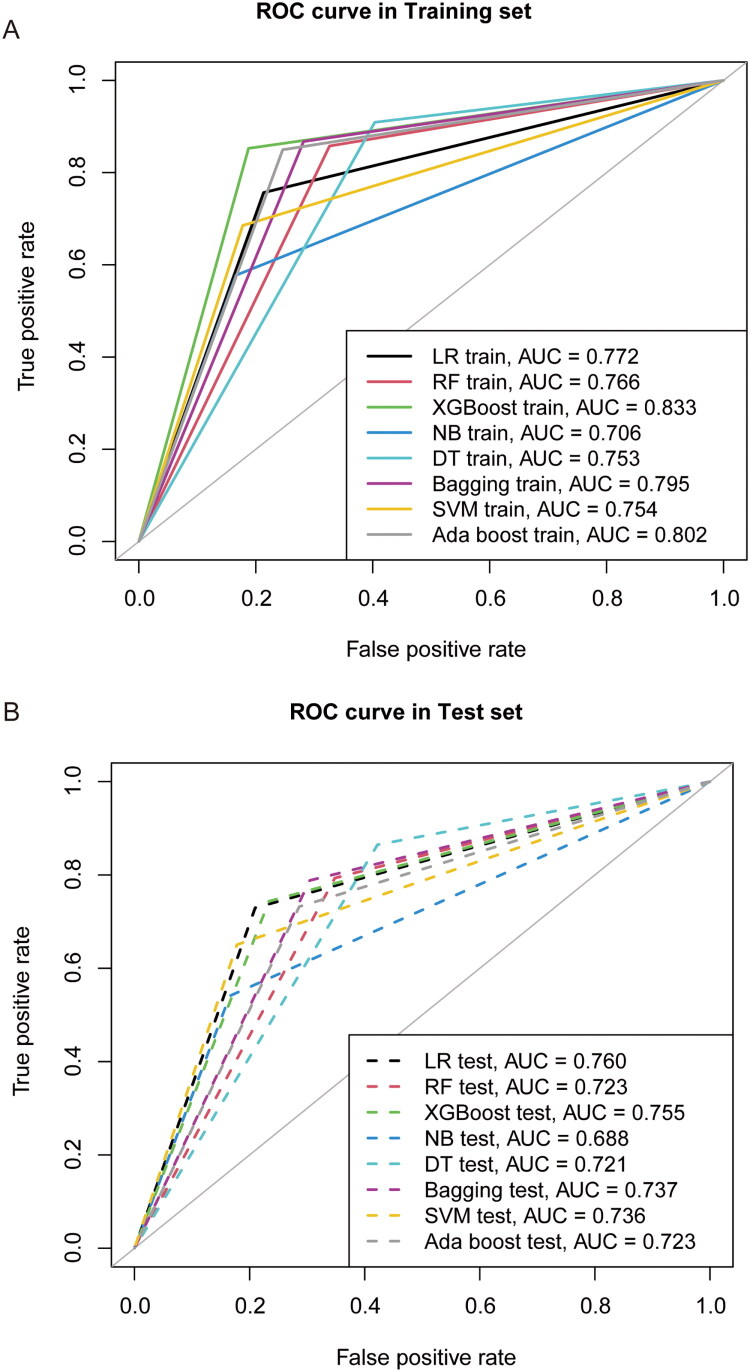
The Receiver operative curve of eight models from the training and test sets. A and B. ROC curves of eight different models with the LASSO selected variables in the training set and validated in the test set. Each model is represented in a unique color, and is consistent throughout the training and test sets.

All evaluation metrics for the training and test sets were AUC and were visualized using the ROC curve. As shown in Figure 4A, we drew ROC curves for different models in various colors. We observed that the highest AUC in the training set was achieved by XGBoost (AUC = 0.833), with the corresponding parameters of map depth = 3, eta = 0.3, nthread = 2, and nrounds = 15. Boosting models performed particularly well on the training set, with AdaBoost ranking second (AUC = 0.802). However, test set results revealed a different pattern: logistic regression yielded the highest AUC (0.760), followed closely by XGBoost (0.755), while AdaBoost demonstrated reduced generalizability (AUC = 0.723). Detailed evaluation metrics, including AUC, accuracy, F1 score, sensitivity, specificity, positive predictive value, and negative predictive value, are reported in Tables 2 and 3 for training and test sets, respectively.

**Table 2.** The sensitivity, specificity, positive predictive value (PPV), and negative predictive values (NPV) for all models in the training set.

	AUC	Accuracy	F1 score	Sensitivity	Specificity	PPV	NPV
Logistic Regression	0.772	0.772	0.778	0.787	0.757	0.77	0.774
Random Forest	0.766	0.764	0.744	0.674	0.858	0.831	0.717
XGBoost	0.833	0.832	0.831	0.812	0.853	0.851	0.814
Naive Bayes	0.706	0.708	0.744	0.834	0.578	0.672	0.77
Decision Tree	0.753	0.750	0.708	0.597	0.909	0.872	0.685
Bagging	0.795	0.795	0.782	0.726	0.866	0.849	0.753
SVM	0.754	0.755	0.774	0.822	0.685	0.73	0.788
AdaBoost	0.802	0.801	0.794	0.754	0.850	0.839	0.769

**Table 3.** The Sensitivity, specificity, positive predictive value (PPV), and negative predictive values (NPV) for all models in the test set.

	AUC	Accuracy	F1 score	Sensitivity	Specificity	PPV	NPV
Logistic Regression	0.76	0.778	0.851	0.79	0.73	0.922	0.461
Random Forest	0.723	0.68	0.766	0.652	0.793	0.928	0.359
XGBoost	0.755	0.762	0.838	0.766	0.744	0.924	0.439
Naive Bayes	0.688	0.777	0.857	0.835	0.541	0.881	0.446
Decision Tree	0.721	0.634	0.717	0.578	0.865	0.946	0.335
Bagging	0.737	0.706	0.789	0.685	0.79	0.93	0.382
SVM	0.736	0.788	0.862	0.822	0.651	0.905	0.474
AdaBoost	0.723	0.717	0.802	0.714	0.732	0.915	0.386

### 3.4. Survival analysis

A summary of patient statistics before discharge is presented in Table 4. The majority of patients recovered from AKI 12 h prior to discharge (*n* = 4130); of the patients who still had AKI prior to discharge, the majority were at Stage 2 AKI (*n* = 596), and the number of patients with Stage 3 AKI (*n* = 315) was slightly higher than that of patients with Stage 2 AKI (*n* = 264). Notably, patients with Stage 3 AKI were younger (mean = 66.9) than those with less severe stages of AKI. Patients who recovered from AKI at the time of discharge were compared to those who did not recover from AKI. The results of this comparison revealed that patients who did not recover from AKI had a higher risk of dying within a one-year period (HR = 1.2, 95% CI = 1.1–1.2), as illustrated in the Supplementary Figures 1A and 1B.

**Table 4.** Summary statistics of the demographic and laboratory tests 12 h before patient discharge.

AKI stages	0	1	2	3	*p* Value
	(*N* = 4,130)	(*N* = 264)	(*N* = 596)	(*N* = 315)	
Sex = M (%)	2,297 (55.6)	137 (51.9)	305 (51.2)	164 (52.1)	0.106
Race (%)					<0.001
ASIAN	130 (3.1)	8 (3.0)	9 (1.5)	4 (1.3)	
BLACK	443 (10.7)	43 (16.3)	68 (11.4)	59 (18.7)	
HISPANIC	111 (2.7)	4 (1.5)	16 (2.7)	15 (4.8)	
OTHER	318 (7.7)	23 (8.7)	44 (7.4)	28 (8.9)	
WHITE	3,128 (75.7)	186 (70.5)	459 (77.0)	209 (66.3)	
Age (mean (*SD*))	69.96 (14.12)	71.06 (13.85)	72.07 (13.13)	66.85 (13.96)	<0.001
Heart rate, bpm (mean (*SD*))	113.32 (24.26)	117.70 (25.17)	118.05 (24.65)	117.61 (25.05)	<0.001
Systolic Blood Pressure, mmhg (mean (*SD*))	161.58 (26.03)	168.41 (30.65)	165.82 (25.65)	172.04 (30.68)	<0.001
Diastolic Blood Pressure, mmhg (mean (*SD*))	103.79 (25.49)	109.88 (31.01)	108.96 (26.16)	112.90 (29.88)	<0.001
Myelin basic protein, mmhg (mean (*SD*))	121.88 (36.22)	129.99 (42.84)	128.86 (39.50)	136.16 (45.88)	<0.001
SBP noninvasive, mmhg (mean (*SD*))	157.27 (24.45)	163.36 (29.53)	161.14 (25.53)	167.26 (30.12)	<0.001
DBP noninvasive, mmhg (mean (*SD*))	101.34 (23.75)	106.09 (28.62)	106.55 (25.68)	110.89 (29.56)	<0.001
MBP noninvasive, mmhg (mean (*SD*))	113.30 (23.93)	117.71 (27.59)	118.10 (25.77)	122.67 (29.12)	<0.001
Respiratory rate, insp/min (mean (*SD*))	32.23 (8.28)	34.26 (8.59)	34.01 (8.38)	36.13 (10.54)	<0.001
Temperature, °C (mean (*SD*))	37.64 (0.79)	37.72 (0.80)	37.77 (0.78)	37.88 (0.84)	<0.001
Simplified Acute Physiology Score II (mean (*SD*))	40.00 (11.90)	44.75 (11.63)	45.58 (12.77)	50.49 (11.44)	<0.001
Hematocrit, % (mean (*SD*))	37.20 (6.02)	37.12 (6.06)	37.32 (5.97)	36.81 (5.94)	0.671
Hemoglobin, g/dL (mean (*SD*))	12.04 (2.07)	11.87 (2.00)	11.98 (1.98)	11.85 (2.11)	0.221
Platelets, K/μL (mean (*SD*))	353.74 (177.26)	346.59 (153.61)	343.74 (180.38)	323.68 (158.40)	0.021
White blood cell, K/μL (mean (*SD*))	16.47 (15.15)	16.08 (8.14)	17.19 (11.88)	21.01 (31.55)	<0 .001
Anion gap, mEq/L (mean (*SD*))	18.82 (4.21)	20.58 (5.71)	19.43 (4.84)	22.99 (6.48)	<0.001
Bicarbonate, mEq/L (mean (*SD*))	30.77 (4.75)	31.61 (5.16)	31.56 (5.47)	31.49 (4.89)	<0.001
Blood urea nitrogen, mg/dL (mean (*SD*))	44.04 (30.88)	58.12 (35.48)	51.83 (32.93)	75.13 (39.96)	<0.001
Calcium, mg/dL (mean (*SD*))	9.47 (1.22)	9.56 (0.97)	9.44 (0.90)	9.72 (1.11)	0.001
Chloride, mEq/L (mean (*SD*))	107.94 (5.85)	107.65 (5.96)	109.15 (6.05)	107.75 (7.06)	<0.001
Creatinine, mg/dL (mean (*SD*))	1.91 (1.86)	3.29 (3.12)	2.27 (1.80)	5.36 (3.63)	<0.001
Glucose, mg/dL (mean (*SD*))	231.66 (207.94)	264.98 (227.89)	257.26 (222.69)	269.13 (194.93)	<0.001
Sodium, mEq/L (mean (*SD*))	143.59 (4.77)	144.01 (4.87)	144.50 (4.81)	144.14 (4.66)	<0.001
Potassium, mEq/L (mean (*SD*))	5.27 (1.07)	5.65 (1.27)	5.39 (1.12)	6.01 (1.37)	<0.001
Absolute Basophils, K/μL (mean (*SD*))	4.16 (15.46)	3.72 (4.58)	4.75 (10.15)	5.55 (18.46)	0.31
Absolute Eosinophils, K/μL (mean (*SD*))	18.45 (41.29)	19.55 (39.17)	22.64 (67.85)	28.31 (93.71)	0.003
Absolute Lymphocytes, K/μL (mean (SD))	144.41 (905.89)	108.02 (154.40)	160.79 (725.58)	121.70 (213.97)	0.815
Absolute Monocytes, K/μL (mean (*SD*))	48.55 (83.15)	44.81 (49.18)	50.33 (54.18)	80.01 (425.07)	<0.001
Absolute Neutrophils, K/μL (mean (*SD*))	654.48 (782.33)	657.91 (739.42)	751.59 (769.04)	758.28 (926.12)	0.007
Basophils, % (mean (*SD*))	0.83 (1.28)	0.76 (0.64)	0.76 (0.79)	0.85 (0.90)	0.404
Eosinophils, % (mean (*SD*))	3.84 (4.56)	4.03 (4.67)	3.88 (4.74)	4.63 (5.42)	0.035
Lymphocytes, % (mean (*SD*))	23.61 (16.71)	22.35 (14.86)	21.64 (15.00)	21.93 (16.37)	0.015
Monocytes, % (mean (*SD*))	10.26 (7.01)	9.86 (5.17)	9.89 (6.63)	10.66 (7.28)	0.327
Neutrophils, % (mean (*SD*))	81.83 (11.65)	82.87 (9.87)	83.34 (10.54)	83.11 (10.62)	0.005
Prothrombin time, sec (mean (*SD*))	22.89 (18.50)	26.96 (19.90)	27.18 (21.65)	32.20 (29.04)	<0.001
Partial thromboplastin time, sec (mean (*SD*))	58.26 (41.39)	71.18 (47.96)	68.92 (46.41)	81.96 (49.08)	<0.001
Survival time, day (mean (*SD*))	97.72 (100.49)	87.45 (95.50)	84.41 (96.75)	76.89 (89.19)	<0.001
Neutrophil-to-lymphocyte ratio (mean (*SD*))	6.10 (7.32)	6.30 (8.02)	7.03 (11.03)	6.62 (6.81)	0.044
Monocyte-to-lymphocyte ratio (mean (*SD*))	0.59 (0.54)	0.61 (0.61)	0.61 (0.54)	0.73 (1.07)	<0.001
Basophils-to-lymphocytes ratio (mean (*SD*))	0.04 (0.04)	0.04 (0.04)	0.04 (0.07)	0.05 (0.14)	<0.001
Eosinophils-to-lymphocytes ratio (mean (*SD*))	0.19 (0.33)	0.21 (0.26)	0.22 (0.32)	0.29 (0.59)	<0.001
Systemic immune-inflammation (mean (*SD*))	2134.02 (2,845.63)	2255.13 (3,942.17)	2216.94 (2,746.40)	2062.90 (2,307.12)	0.782

**Figure 5.**
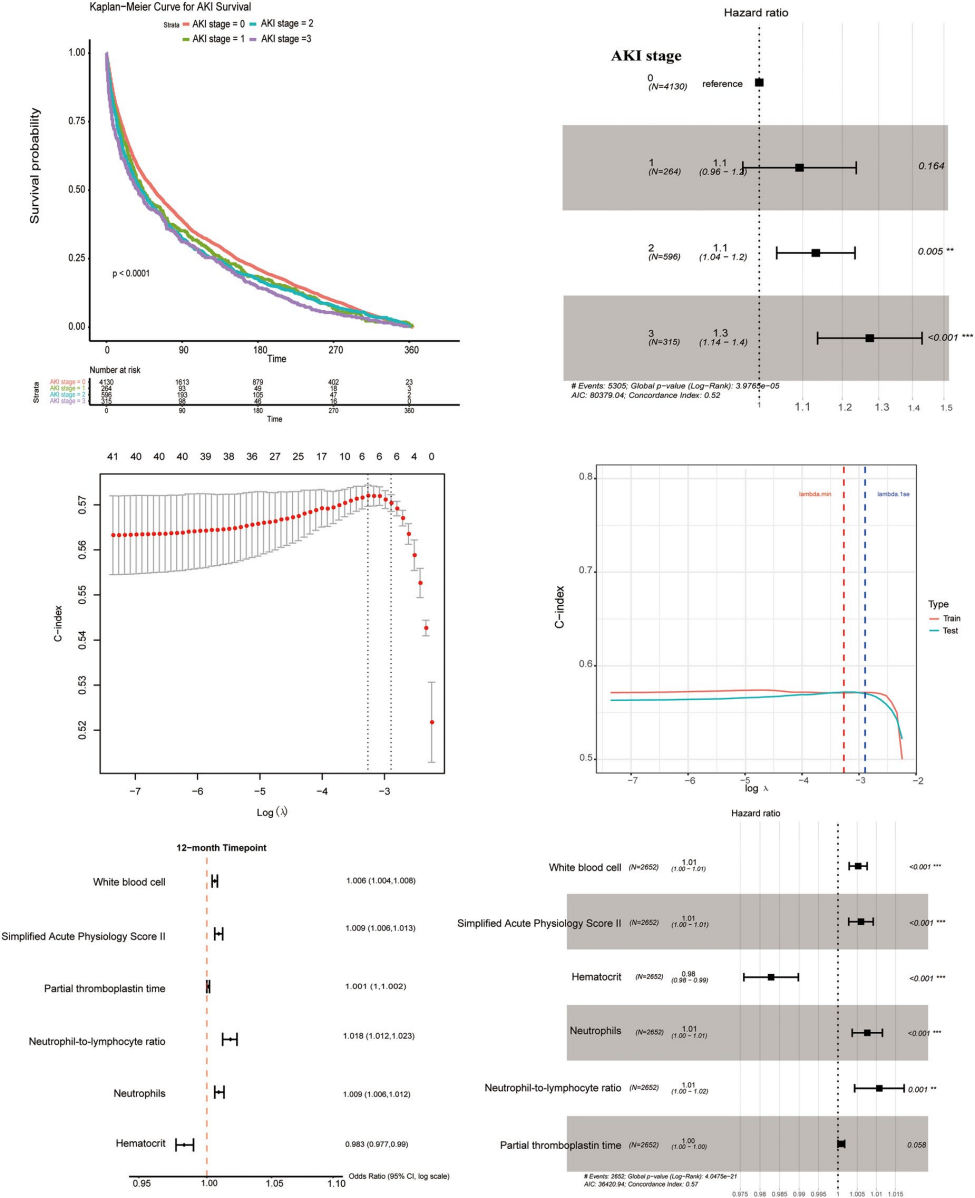
Survival analysis of patients previously diagnosed with AKI. (A) Kaplan-meier curve of patients discharged from hospital based on the AKI stages measured 12 h before discharge. (B) Forest plot of hazard ratios of patients at different AKI stages before discharge. (C) Plot indicating the minimum value of lambda (the left dashed line) with corresponding number of features determined using Cox-LASSO regression. (D) Line plot showing the c-index of the cox proportional hazard model in training and test sets. (E) Forest plot of univariate Cox-LASSO regression selected survival related variables and their corresponding hazards ratios. (F) Forest plot of multivariate Cox-LASSO regression selected survival related variables and their corresponding hazards ratios.

First, we compared the 1-year survival of patients who were discharged from the hospital based on their AKI stage and observed significant differences among the four groups. As expected, the Kaplan-Meier curve revealed that the lower the AKI stage, the higher the survival rate during the year (Figure 5A). The 90-day survival rate for all groups was < 50%, and none of the patients with Stage 3 AKI survived for 1 year. We then ran a univariate Cox proportional hazard model on the survival data of patients with different AKI stages, and the results were in accordance with those of the Kaplan-Meier curve; the risk of death was higher in patients with later AKI stages than in those without AKI (Figure 5B). Treating patients without AKI as the reference group, patients with Stage 2 AKI had higher risk of death than the reference (hazard ratio [HR] = 1.1, 95% confidence interval [CI]: 1.04–1.20); the risk of death for patients in Stage 3 AKI were even higher (HR = 1.3. 95% CI: 1.14–1.40).

Furthermore, we used LASSO regression to investigate which variables predicted the survival rate after discharge based on the information gathered 12 h prior to discharge. With the dot plot, we observed that the best lambda value was around log (−3), with an optimal number of variables of six, according to the left dotted line (Figure 5C). Moreover, we tested the model using six variables with the C-index metric, which was approximately 0.57 for both the training and test sets (Figure 5D).

To further evaluate these six LASSO-selected variables, we performed univariate Cox proportional hazards analyses, visualized in a forest plot (Figure 5E). The six variables were independent prognostic predictors from the univariate Cox-LASSO regression. The independent predictors selected from Cox-LASSO were white blood cells (WBC) (HR = 1.006, 95% CI: 1.004–1.006), SAPS II score (HR = 1.009, 95% CI: 1.009–1.013), PTT (HR = 1.001, 95% CI = 1.000–1.002), NLR (HR = 1.018, 95% CI = 1.012–1.023), neutrophils (HR = 1.009, 95% CI: 1.006–1.012) and hematocrit (HR = 0.983, 95% CI: 0.977–0.990). From the forest plot, we observed that all the six variables, except for hematocrit, had an HR greater than 1, indicating that the higher the value of the risk factor, the greater the risk of death. For hematocrit, an HR less than 1 indicated a decreased risk of death with higher hematocrit values. The NLR results showed the greatest risk of death in the patients with higher NLR. For further exploration, we performed multivariate Cox PH regression on the selected variables to determine whether patient survival changed after adjusting for other variables (Figure 5F). After adjusting for covariates, WBC (HR = 1.01, 95% CI: 1.00–1.01), SAPS II score (HR = 1.01, 95% CI: 1.00–1.01), hematocrit (HR = 0.98, 95% CI: 0.98–0.99), neutrophils (HR = 1.01, 95% CI: 1.00–1.01), and NLR (HR = 1.01, 95% CI: 1.00–1.02) were significantly associated with the long-term mortality. These significant variables were selected as independent risk factors for 1-year mortality after discharge in patients with AKI.

## 4. Discussion

This study primarily aimed to predict in-hospital mortality in AKI patients using laboratory tests collected within 24 h of admission. Among eight machine learning algorithms evaluated, XGBoost demonstrated superior performance—consistent with prior findings in cardiac surgery-associated AKI populations [36]. Despite the slight difference between the training AUC in XGBoost and AdaBoost, XGBoost was superior to AdaBoost in terms of computing speed. Furthermore, although the test AUC of the logistic regression was marginally higher than that of XGBoost, the visual presentation of the variable importance of XGBoost was superior. Concurrently, the AUC of the logistic regression in the test set was comparable to that of XGBoost. Consequently, logistic regression is also an excellent model for those interested in understanding the contribution of each predictor, given its superior interpretability. This machine learning algorithm allowed us to gain insights into prospective features that may be associated with in-hospital mortality in patients with AKI.

The secondary objective of this study was to identify risk predictors of adverse outcomes in AKI patients after hospital discharge. Unlike previous studies that used laboratory tests from samples obtained with 24-h of ICU admission, we instead extracted parameters measured 12h prior to discharge and hoped that they could yield better results [29,37,38]. The Kaplan-Meier curve was employed to observe the survival rate of patients discharged from hospital care, revealing that this rate was less than 50% for all groups at day 90. This result was consistent with a study from Sudan that reported that the mortality of patients with AKI was 31.2%, and another study indicated that patients with any form of AKI had a 50% higher risk of death than those without AKI [39,40]. These findings highlight the persistently poor post-discharge prognosis in AKI patients and underscore the need to identify actionable prognostic indicators to improve outcomes.

The SAPS II score measures the acute physiology scores of patients on the first day of ICU admission and was demonstrated to be the most important variable in the random forest, XGBoost, and decision trees in our study. The SAPS II score has been demonstrated to possess satisfactory predictive capabilities in models of acute kidney injury (AKI) mortality, particularly in the context of sepsis-associated AKI [41,42]. This suggests that, rather than identifying a single predictor of laboratory test results, a comprehensive indicator reflecting multiple physiological features of patients may be a more reasonable approach. In addition to SAPS II, other key features identified by XGBoost and random forest included heart rate, prothrombin time (PT), hematocrit, anion gap, and NLR. Unlike composite scores, these individual biomarkers may reflect specific pathological mechanisms, such as coagulation dysfunction or systemic inflammation. This shifts the utility of machine learning models from mere risk alert systems to tools that can potentially guide targeted interventions.

Given the importance of these variables, we recommend their measurement using rapid bedside diagnostic tools, such as hand-held hematocrit meters or point-of-care blood gas analyzers, which enable results within minutes. Looking ahead, we propose developing a dynamic, continuously updating risk-scoring model incorporating these features. This model would refresh hourly, and once a predefined risk threshold is surpassed, it would trigger an automated alert to designated clinical staff, enabling timely intervention and potentially improving outcomes in AKI patients.

Among immune cells included as predictors in the model, monocytes were the most important variables in XGBoost. A recent study identified a potential biomarker for the treatment of monocyte infiltration in sepsis-associated AKI using weighted gene co-expression network (WGCNA) and immune cell infiltration analyses [43]. Furthermore, using survival analysis, we identified six independent survival-related predictors for long-term all-cause mortality based on laboratory tests on samples obtained 12-h prior to patient discharge. The results of this study demonstrated a strong correlation between neutrophil count and long-term mortality in patients previously diagnosed with AKI.

Surprisingly, several immune cell-to-lymphocyte ratios were not retained in the final models for either in-hospital or post-discharge mortality. While previous research have reported that immune cell ratios such as the NLR ratio and monocyte-to-lymphocyte ratio (MLR) are positively associated with 30-day and 90-day mortality in AKI [44,45], these markers were not selected in our models. This discrepancy may reflect timing, patient heterogeneity, or variable thresholds. Nevertheless, immune cells remain critical components of both short- and long-term mortality risk stratification in AKI patients. Thus, their dynamic monitoring should be integrated into both inpatient care and post-discharge follow-up.

The correlation observed between the identified risk elements and 1-year mortality indicates that medical practitioners should consider these biochemical indicators, particularly in the post-discharge context. In addition, most previously published studies used laboratory tests within 24 h in the ICU when searching for associations between predictors and 30-day, 90-day, and 1-year mortality analyses [37,46,47]. In contrast, we argue that discharge-phase laboratory data may offer more clinically actionable prognostic insights. To our knowledge, this is the first study to systematically evaluate the prognostic value of laboratory results obtained 12 h prior to hospital discharge in AKI patients. These findings underscore the need for further research into the timing and clinical utility of discharge laboratory testing.

However, this study has several limitations that must be addressed. First, because this study was based on retrospective clinical data, we were unable to draw causal inferences or mitigate the generalizability of our findings. Second, the estimated glomerular filtration rate (eGFR), a common variable for diagnosing AKI, was not included because of the high proportion of missing data in our study population. Moreover, AKI is one of the most prevalent comorbidities of sepsis in hospitalized patients. However, the present study did not incorporate subgroup analyses that restricted the patient cohort to those with sepsis, alcohol use disorder, or patients who underwent cardiac surgery, because these conditions exhibit greater disease specificity [48–50]. Notwithstanding the remarkable efficacy of machine learning algorithms in predicting the mortality of patients with AKI, the research presented by Wu et al. highlighted the disparities in the performance of these algorithms in patients of varying racial, geographical, and social economic backgrounds [51]. Consequently, it is imperative to conduct subgroup analyses to ensure the efficacy of machine learning (ML) in predicting mortality from AKI and other diseases. Future studies should refine the range of patients selected from the dataset to achieve a more specific AKI prognosis. In addition, the cause of death was not available in the dataset; however, we believe that this will play an essential role in making more precise predictions.

## 5. Conclusion

Among hospitalized patients with AKI, the SAPS II score, heart rate, and body temperature emerged as the most significant predictors of in-hospital mortality. Our findings demonstrate that machine learning algorithms can effectively support clinical decision-making in this population. Furthermore, Cox regression analysis identified six independent post-discharge risk factors, emphasizing the importance of monitoring immune cell profiles during follow-up. Future studies should validate the impact of these predictors on long-term survival and incorporate them into dynamic risk stratification tools for AKI.

## Supplementary Material

Supplemental Material
