# Supplementary figures and images for: Prediction of all-cause in-hospital mortality after ICU admission and 1-year mortality after discharge of patients with acute kidney injury
Source: Ren Fail. 2025 Sep 29;47(1):2562445. doi: 10.1080/0886022X.2025.2562445 (PMC12481539; doi:10.1080/0886022X.2025.2562445)

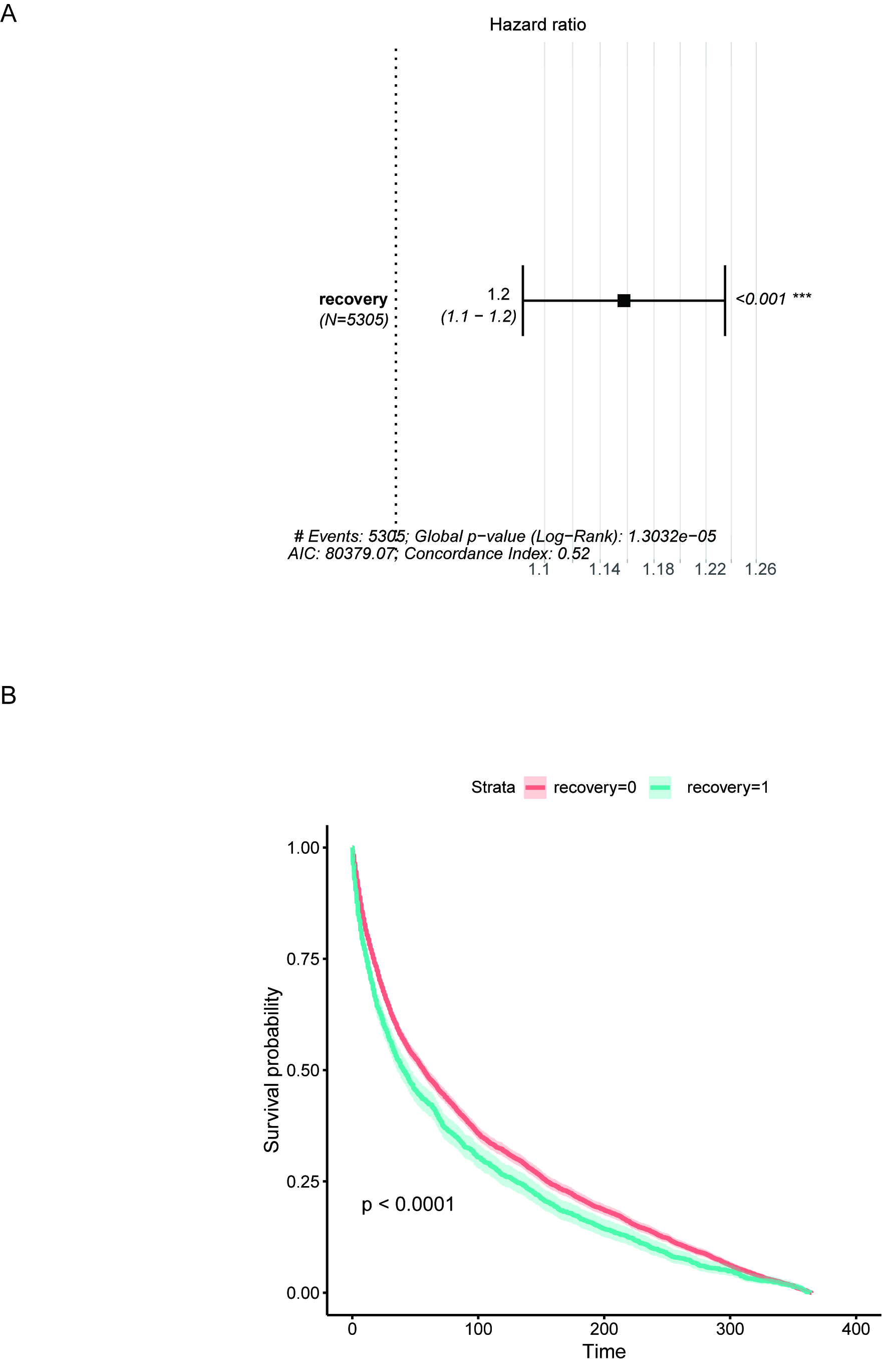

Supplement: Supplemental Material [file IRNF_A_2562445_SM7325.tif]
